# Early distinction of lymph node metastasis in patients with soft tissue sarcoma and individualized survival prediction using the online available nomograms: A population-based analysis

**DOI:** 10.3389/fonc.2022.959804

**Published:** 2022-12-07

**Authors:** Yuexin Tong, Yangwei Pi, Yuekai Cui, Liming Jiang, Yan Gong, Dongxu Zhao

**Affiliations:** ^1^ Department of Orthopedics, The China-Japan Union Hospital of Jilin University, Changchun, Jilin, China; ^2^ The Second Clinical Medical School of the Wenzhou Medical University, Wenzhou, Zhejiang, China

**Keywords:** lymph node metastasis, soft tissue sarcoma, nomogram, overall survival, cancer specific survival, SEER

## Abstract

**Background:**

The presence of metastatic tumor cells in regional lymph nodes is considered as a significant indicator for inferior prognosis. This study aimed to construct some predictive models to quantify the probability of lymph node metastasis (LNM) and survival rate of patients with soft tissue sarcoma (STS) with LNM.

**Methods:**

Research data were extracted from the Surveillance, Epidemiology, and End Results (SEER) database between 2004 and 2017, and data of patients with STS from our medical institution were collected to form an external testing set. Univariate and multivariate logistic regression analyses were used to determine the independent risk factors for developing LNM. On the basis of the identified variables, we developed a diagnostic nomogram to predict the risk of LNM in patients with STS. Those patients with STS presenting with LNM were retrieved to build a cohort for identifying the independent prognostic factors through univariate and multivariate Cox regression analysis. Then, two nomograms incorporating the independent prognostic predictors were developed to predict the overall survival (OS) and cancer-specific survival (CSS) for patients with STS with LNM. Kaplan–Meier (K-M) survival analysis was conducted to study the survival difference. Moreover, validations of these nomograms were performed by the receiver operating characteristic curves, the area under the curve, calibration curves, and the decision curve analysis (DCA).

**Results:**

A total of 16,601 patients with STS from the SEER database were enrolled in our study, of which 659 (3.97%) had LNM at the initial diagnosis. K-M survival analysis indicated that patients with LNM had poorer survival rate. Sex, histology, primary site, grade, M stage, and T stage were found to be independently related with development of LNM in patients with STS. Age, grade, histology, M stage, T stage, chemotherapy, radiotherapy, and surgery were identified as the independent prognostic factors for OS of patients with STS with LNM, and age, grade, M stage, T stage, radiotherapy, and surgery were determined as the independent prognostic factors for CSS. Subsequently, we constructed three nomograms, and their online versions are as follows: https://tyxupup.shinyapps.io/probabilityofLNMforSTSpatients/, https://tyxupup.shinyapps.io/OSofSTSpatientswithLNM/, and https://tyxupup.shinyapps.io/CSSofSTSpatientswithLNM/. The areas under the curve (AUCs) of diagnostic nomogram were 0.839 in the training set, 0.811 in the testing set, and 0.852 in the external testing set. For prognostic nomograms, the AUCs of 24-, 36-, and 48-month OS were 0.820, 0.794, and 0.792 in the training set and 0.759, 0.728, and 0.775 in the testing set, respectively; the AUCs of 24-, 36-, and 48-month CSS were 0.793, 0.777, and 0.775 in the training set and 0.775, 0.744, and 0.738 in the testing set, respectively. Furthermore, calibration curves suggested that the predicted values were consistent with the actual values. For the DCA, our nomograms showed a superior net benefit across a wider scale of threshold probabilities for the prediction of risk and survival rate for patients with STS with LNM.

**Conclusion:**

These newly proposed nomograms promise to be useful tools in predicting the risk of LNM for patients with STS and individualized survival prediction for patients with STS with LNM, which may help to guide clinical practice.

## Introduction

Soft tissue sarcoma (STS) refers to a group of uncommon mesenchymal malignancies, only accounting for approximately 1% of all solid tumors (1). In recent years, three mainstream treatments for patients with STS, namely, surgery, radiotherapy, and chemotherapy, have shown progressive effects (2, 3). Unfortunately, the prognosis of patients diagnosed with STS remains unsatisfactory, owing to the local recurrence and metastases disease (4).

Compared with the most common lung metastases, lymph node metastasis (LNM) is a more rare but equally vital prognostic factor, with the overall prevalence varying from 1.7% to 5.9% (5). Despite the low incidence, the confirmation of LNM seems to be a clinical expression of the biological aggressiveness of the STS, patients with LNM are prone to have unsatisfactory survival outcome (6). The 5-year survival for patients with STS with LNM is reported to range from 12.5% to 45.5% (7, 8), similar to the 5-year overall survival (OS) rate in patients with oligometastatic disease localized to the lungs, which is estimated to be approximately 25% to 40% (9). Because of the dismal prognosis, patients with LNM would be considered to be in the advanced stage of the disease. Hence, the eighth edition staging system of the American Joint Committee on Cancer (AJCC) categorizes these cases as clinical stage IV (10). In this regard, it is important to accurately identify which patients with STS at high risk for LNM. Several previous reports have identified several variables related with the development of LNM, including histological type, tumor grade, primary tumor size, and age (5, 7, 11). Despite a recent article that paid attention to the same topic, they were also focused on exploring risk factors (12). Nevertheless, assessing the probability of a certain clinical outcome by a few single variables alone is inaccurate, which had been demonstrated in several previous studies (13–15). The lack of comprehensive predictive model means that the probability of LNM in patients with STS and of survival rate in patients with STS with LNM still cannot be quantified.

Given the low incidence of LNM in STS, it is relatively difficult to acquire enough positive research cases in a single institution. The Surveillance, Epidemiology, and End Results (SEER) database is the largest publicly available dataset, which collects data from 18 cancer registries and involves about 30% of the US population (16). In this regard, the SEER database is considered to be a reliable source of research data for investigating such rare cancers. Therefore, using the population-based data, the objective of this study is to construct three web-based nomograms to predict the probability of LNM in patients diagnosed with STS and the survival rate of patients with STS with LNM.

## Materials and methods

### Patients and selected criteria

**Figure 1:**
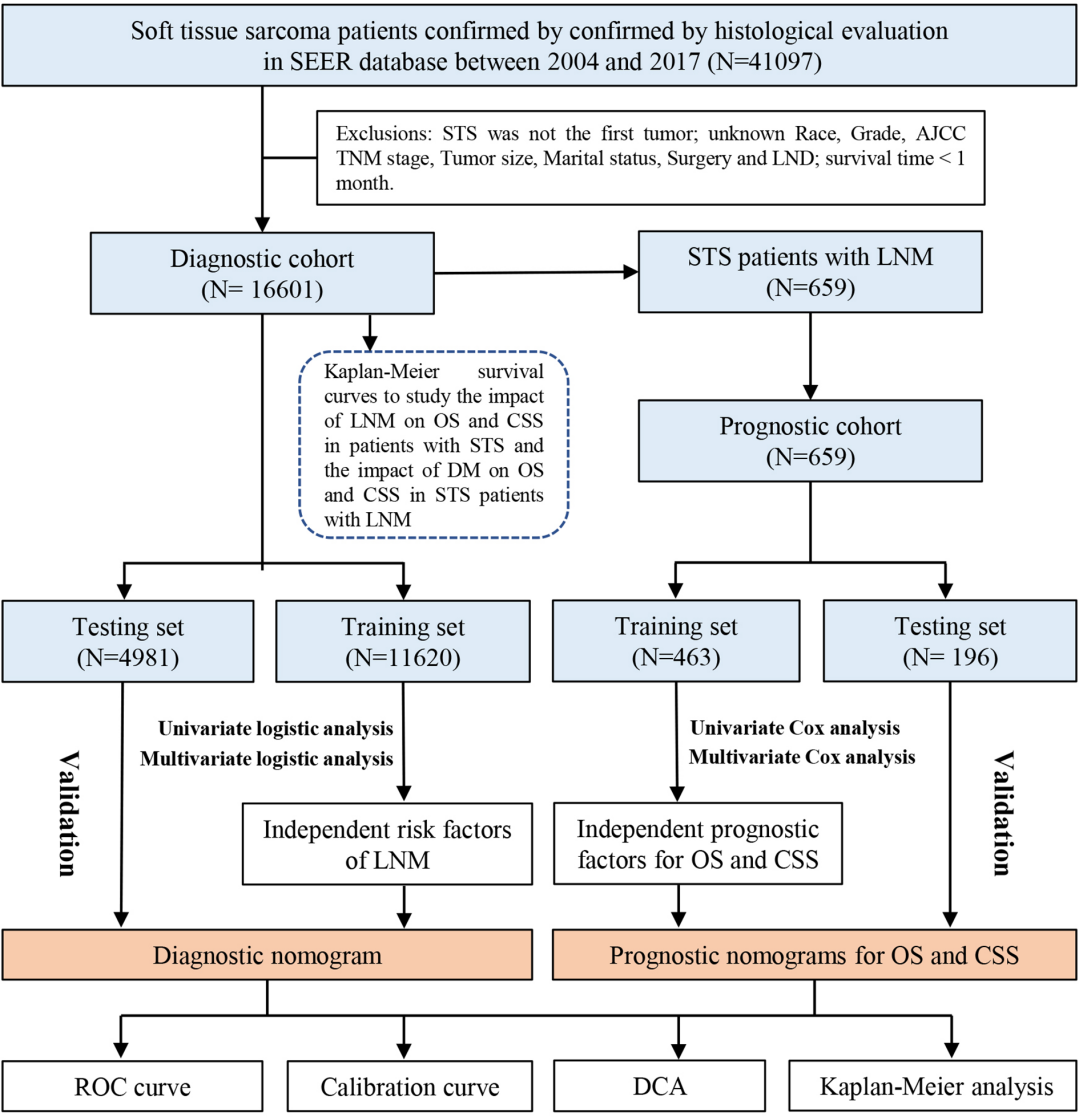
The detailed selection process of patients and workflow of study.

All the information of patients diagnosed with STS was extracted from the SEER database by SEER * Stat software version 8.4.0. The inclusion criteria of our study sample were as follows (1): patients diagnosed with STS between 2004 and 2017; and (2) patients were diagnosed by histological confirmation. In addition, the exclusion criteria were as follows: (1) STS was not the first malignant tumor and more than one primary tumor; (2) patients for whom the following information was unknown, including age, race, sex, histology, grade, stage TNM, marital status, surgery, and lymph node dissection (LND); and (3) the survival time less than 1 month. All of the eligible patients with STS were used to form the diagnostic study cohort and those patients with STS with LNM were included in the prognostic study cohort. In this study, TNM staging was transferred to the eighth edition of the AJCC/UICC TNM staging system. The detailed selection process of patients and the workflow of study are shown in **Figure 1**. In addition, the external testing set was obtained from China-Japan Union Hospital of Jilin University. Two pathologists were selected to perform the pathological evaluation of the patient’s biopsy specimens or pathological examination report using a blinded method. Moreover, all visit records were selected from the hospital’s electronic medical record system. For patients lacking medical records, as long as they can provide such information in outside institutions, they are still considered to be eligible for inclusion. All methods were performed in accordance with the relevant guidelines and regulations (Declaration of Helsinki).

### Study variables

On the basis of the clinicopathological characteristics provided in the SEER database and published literatures, the following eight variables were collected to identify the independent LNM-related risk factors, including age, sex, race, histology, grade, M stage, T stage and primary site. In addition, other four treatment variables (chemotherapy, LND, radiotherapy and surgery) and one sociological variable (marital status) were collected to identify the independent prognostic factors for patients with STS with LNM. In these variables, grade was classified into well differentiation (I–II) and poor differentiation (III–IV). The primary site included extremity, trunk, and head and neck. Moreover, quantitative data were classified as categorical data, including non-elderly patients and elderly patients (<65 and ≥65 years). OS, the time between the date of disease diagnosis and the date of death from any reasons, was determined to be one of the endpoint of this study. In addition, considering that cancer-specific survival (CSS) was, to some extent, more closely related to tumor-mediated patient prognosis, it might provide more precise guidance for the treatment of patients with STS with LNM. CSS was defined as another study endpoint.

### Statistical analysis

All selected patients with STS and patients with STS with LNM were both randomly divided into training sets and testing sets in a ratio of 7:3 approximately. The chi-square test was used to compare differences between the training set and the testing set. The training sets were used to construct and validate nomograms, and the testing sets were used to validate nomograms. In the part of construction and validation of the diagnostic nomogram, the external testing set was formed for the external validation using the information from another regional population to assess the extrapolation of the model. First, the Kaplan–Meier (K-M) survival analysis with the log-rank test was performed to compare the survival probability between patients with STS with LNM and without LNM and investigate differences of OS and CSS between LNM-positive patients with and those without distant metastasis (DM). Then, univariate and multivariate logistic regression analyses were used to explore independent LNM-related risk factors in patients with STS in diagnostic study cohort. The relevance between predictors and LNM was shown by odd ratios and corresponding 95% confidence intervals (CIs). The correlation between clinicopathological characteristics and survival rate of patients with STS with LNM was estimated by univariate and multivariate Cox analyses. The hazard ratios and corresponding 95% CIs were calculated. Finally, on the basis of the results of logistic analyses and Cox analyses, the diagnostic nomogram and prognostic nomograms were constructed by “rms” package in R software, respectively. Meanwhile, three corresponding web-based calculators were further established on the basis of the nomograms using the “Dynnom” package.

The receiver operating characteristic (ROC) curves and C-index values were used to evaluate the discrimination of the nomogram. The higher value of area under the curves (AUC) indicated better discrimination. The calibration curve was carried out to evaluate the consistence between actual outcomes and predicted outcomes. In addition, the decision curve analysis (DCA) curves were performed to calculate the net benefit of the predictive model, which can show the clinical practicability. In addition, a k-fold (k = 10) cross-validation method was performed to validate prognostic nomograms.

Furthermore, we calculated the total score of each patient with STS with LNM using these prognostic nomograms, and they were divided into two subgroups based on the median total score. The K-M survival analysis with the log-rank test was performed to evaluate the ability in stratifying patients with STS with LNM according to the risk of mortality. Statistical analysis and mapping involved in our study were conducted in SPSS 26.0 and R software version 4.0.2 (https://www.r-project.org/). P-value <0.05 (both sides) was regarded as statistically markedly.

## Results

### Basic characteristics of patients with STS with or without LNM

According to our inclusion and exclusion criteria, a total of 16,601 patients with STS were finally included in this study. We also collected information on 181 cases of patients with STS from our medical institution. Together, they formed the diagnostic study cohort, which was used to identify the independent risk factors for LNM in patients with STS and then construct a diagnostic nomogram. They were divided into a training set (N = 11,620), a testing set (N = 4,981), and an external testing set (N = 181). As described in **Table 1**. Most patients were white (80.11% in the training set and 80.24% in the testing set). Patients in the <65-year age group (63.18% in the training set and 63.88% in the testing set) made up the majority of the study sample. The male-to-female ratio was close to 1:1. The most common primary site was extremity (59.65% in the training set and 59.12% in the testing set). According to the AJCC TNM staging system, in the overall cohort, nearly one-third of the patients were staged as T1 (N = 3465, 29.82%) and T2 (N = 1437, 28.85%). Up to 90.64% of patients did not have metastatic diseases (M0 stage) at the time of initial diagnosis.

**Table 1:** The demographic and clinicopathological characteristics of patients with soft tissue sarcoma with or without lymph node metastasis.

Variables		Overall cohort from SEER (N = 16,601, %)	Training set (N = 11,620, %)	Testing set (N = 4981, %)	Externaltesting set (N = 181, %)	P-value^#^
**Age**	**<65 years**	10,524 (63.39)	7,342 (63.18)	3,182 (63.88)	134 (74.00)	0.4016
	**≥65 years**	6,077 (36.61)	4,278 (36.82)	1,799 (36.12)	47 (26.00)	
**Sex**	**Female**	7,339 (44.21)	5,147 (44.29)	2,192 (44.01)	79 (43.60)	0.7457
	**Male**	9262 (55.79)	6,473 (55.71)	2,789 (55.99)	102 (56.40)	
**Race**	**Black**	1,814 (10.93)	1,237 (10.65)	577 (11.58)	181 (100.00)	0.0559
	**Other**	1,481 (8.92)	1,074 (9.24)	407 (8.17)	0 (0.00)	
	**White**	13,306 (80.15)	9,309 (80.11)	3,997 (80.24)	0 (0.00)	
**Primary site**	**Extremity**	9,876 (59.49)	6,931 (59.65)	2,945 (59.12)	72 (39.80)	0.4018
	**Trunk**	5,829 (35.11)	4,049 (34.85)	1,780 (35.74)	99 (54.70)	
	**Head and neck**	896 (5.40)	640 (5.51)	256 (5.14)	10 (5.50)	
**Histology**	**Fibrosarcoma**	3,121 (18.80)	2,168 (18.66)	953 (19.13)	31 (17.10)	0.3823
	**Liposarcoma**	3,822 (23.02)	2,652 (22.82)	1,170 (23.49)	57 (31.5)	
	**Leiomyosarcoma**	2,066 (12.45)	1,462 (12.58)	604 (12.13)	12 (6.60)	
	**Synovival sarcoma**	864 (5.20)	598 (5.15)	266 (5.34)	14 (7.70)	
	**MPNST**	624 (3.76)	441 (3.80)	183 (3.67)	20 (11.00)	
	**Rhabdomyosarcoma**	363 (2.19)	237 (2.04)	126 (2.53)	9 (5.00)	
	**Hemangiosarcoma**	397 (2.39)	281 (2.42)	116 (2.33)	10 (5.50)	
	**Other**	5,344 (32.19)	3,781 (32.54)	1,563 (31.38)	28 (15.50)	
**Grade**	**Well differentiation**	3,203 (19.29)	2,241 (19.29)	962 (19.31)	83 (45.90)	0.1259
	**Poor differentiation**	3,183 (19.17)	2,225 (19.15)	958 (19.23)	98 (54.10)	
**T stage**	**T1**	4,902 (29.53)	3,465 (29.82)	1,437 (28.85)	54 (29.80)	0.2251
	**T2**	5,296 (31.90)	3,710 (31.93)	1,586 (31.84)	61 (33.70)	
	**T3**	3,125 (18.82)	2,142 (18.43)	983 (19.73)	46 (25.40)	
	**T4**	3,278 (19.75)	2,303 (19.82)	975 (19.57)	20 (11.00)	
**N stage**	**N0**	15,942 (96.03)	11,170 (96.13)	4,772 (95.80)	175 (96.70)	0.3501
	**N1**	659 (3.97)	450 (3.87)	209 (4.20)	6 (3.30)	
**M stage**	**M0**	1,5047 (90.64)	10,520 (90.53)	4,527 (90.89)	132 (72.90)	0.4939
	**M1**	1,554 (9.36)	1,100 (9.47)	454 (9.11)	49 (27.10)	

^#^The result of P value was derived from the chi-square test for training set and testing set.

### The impact of LNM on the survival and independent risk factors for LNM in patients with STS

**Figure 2:**
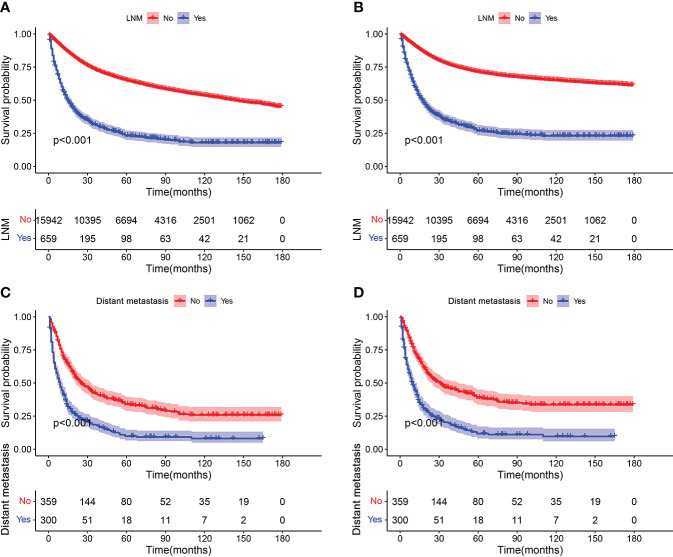
Kaplan–Meier survival analyses to investigate the impact of LNM on OS **(A)** and CSS **(B)** in patients with STS. In addition, survival curves showed that the presence of DM resulted in poorer OS **(C)** and CSS **(D)** in the group of patients with LNM.

K-M survival analysis was used to compare OS and CSS survival probability between patients with STS with LNM and without LNM. The results showed that the OS and CSS rates of patients with STS with LNM were greatly worse than that of patients without LNM (**Figures 2A, B**). We also calculated OS and CSS from 1 to 5 years for both patients with STS with LNM and those without LNM and summarized these results in **Table 2**. Furthermore, because DM was common in patients with STS with LNM (approximately 45.52% of the cases in this cohort), we further compared the discrepancies of prognosis between LNM-positive patients with and without DM, and the survival curves confirmed that the presence of DM always resulted in poorer OS (C) and CSS (D) in patients with STS with LNM (**Figures 2C, D**). According to the univariate logistic regression analysis, some factors were found to be associated with the occurrence of LNM, such as sex, histology, primary site, grade, M stage, and T stage (P < 0.05). Then, on the basis of the above result, multivariate logistic regression was applied to finally determine the independent risk factors, including sex, histology, primary site, grade, M stage, and T stage (P < 0.05). The results are shown in **Table 3**.

**Table 2:** Overall survival and cancer-specific survival within 5 years in patients with STS with and without LNM.

	Patients with STS with LNM	Patients with STS without LNM
1-yeas OS	55.7%	88.8%
2-year OS	38.5%	79.7%
3-year OS	31.5%	72.9%
4-year OS	27.2%	68.7%
5-year OS	23.0%	65.1%
1-year CSS	59.1%	90.5%
2-year CSS	41.6%	82.8%
3-year CSS	34.5%	77.4%
4-year CSS	31.1%	73.9%
5-year CSS	27.0%	71.3%

OS, overall survival; CSS, cancer-specific survival.

**Table 3:** Univariate and multivariate logistic analysis to determine the independent risk factors of lymph node metastasis in patient with soft tissue sarcoma.

Variables	Univariate analysis			Multivariate analysis		
	OR	95% CIs	P-value	OR	95% CIs	P-value
**Age**						
<65 years	Reference					
≥65 years	0.87	0.71–1.06	0.177			
**Sex**						
Female	Reference			Reference		
Male	1.45	1.19–1.76	<0.001	1.38	1.12–1.71	0.002
**Race**						
Black	Reference					
Other	0.88	0.59–1.32	0.549			
White	0.81	0.61–1.07	0.141			
**Primary site**						
Extremity	Reference			Reference		
Trunk	1.8	1.48–2.2	<0.001	1.48	1.19–1.84	<0.001
Head and neck	2.9	2.1–4	<0.001	3.14	2.17–4.53	<0.001
**Histology**						
Fibrosarcoma	Reference			Reference		
Liposarcoma	0.53	0.32–0.89	0.015	0.61	0.36–1.03	0.065
Leiomyosarcoma	1.42	0.89–2.24	0.139	0.98	0.61–1.58	0.940
Synovival sarcoma	2.75	1.68–4.53	<0.001	2.03	1.21–3.39	0.007
MPNST	2.25	1.26–4.02	0.006	1.45	0.79–2.65	0.228
Rhabdomyosarcoma	11.73	7.37–18.67	<0.001	4.86	2.96–7.98	<0.001
Hemangiosarcoma	6.45	3.91–10.65	<0.001	3.61	2.11–6.16	<0.001
Other	3.73	2.64–5.28	<0.001	2.39	1.67–3.41	<0.001
**Grade**						
Well differentiation						
Poor differentiation	5.48	4.05–7.41	<0.001	2.6	1.89–3.57	<0.001
**T stage**						
T1	Reference			Reference		
T2	1.94	1.47–2.56	<0.001	1.55	1.15–2.09	0.004
T3	2.34	1.74–3.15	<0.001	1.76	1.26–2.45	0.001
T4	2.16	1.61–2.91	<0.001	1.77	1.26–2.47	0.001
**M stage**						
M0	Reference			Reference		
M1	10.74	8.82–13.08	<0.001	6.79	5.47–8.42	<0.001

MPNST, malignant peripheral nerve sheath tumor; OR, odds ratio; CIs, confidence intervals.

### Construction and validation of the diagnostic nomogram

**Figure 3:**
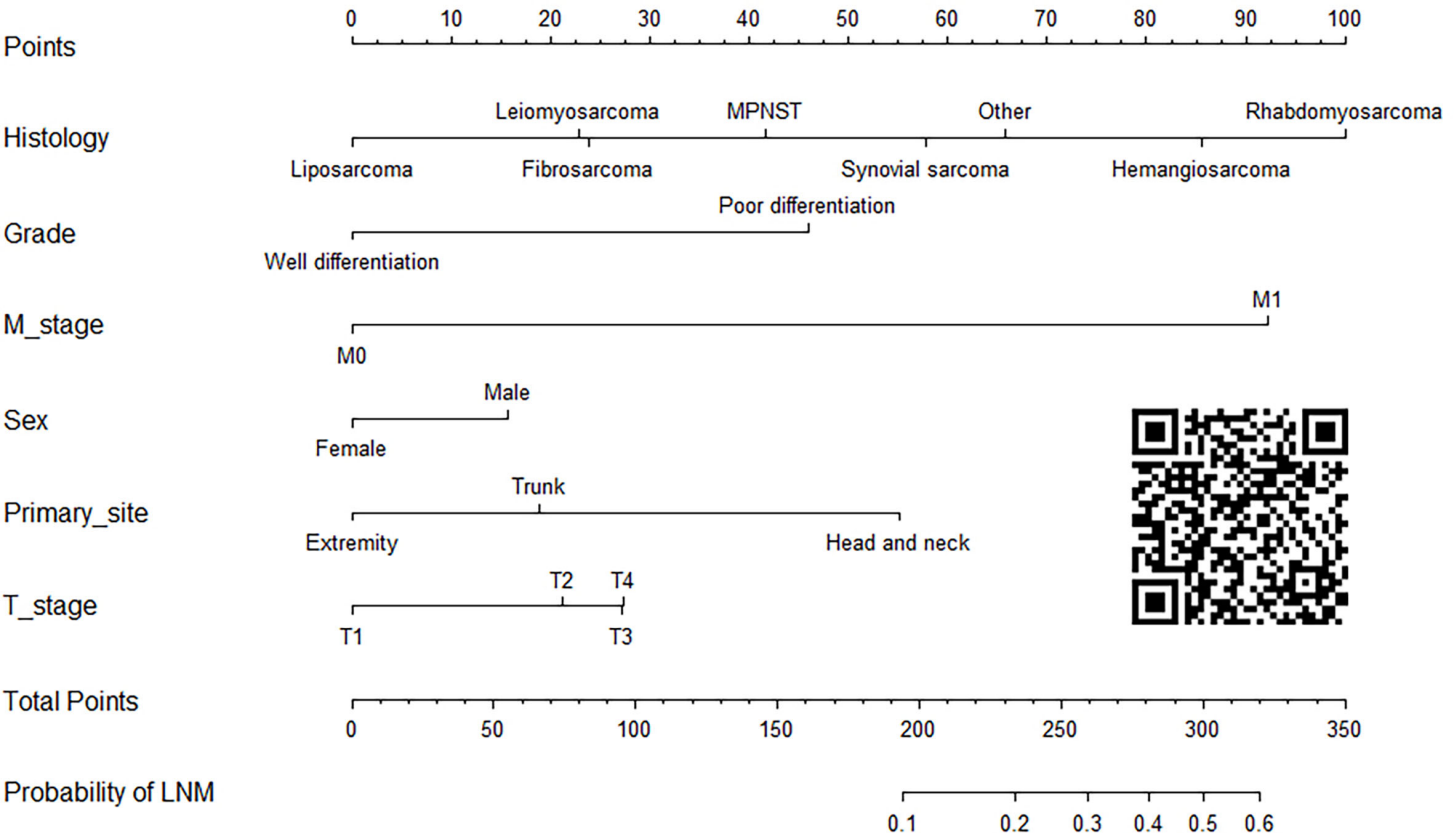
A diagnostic nomogram for quantifying the probability of LNM in patients with STS.

**Figure 4:**
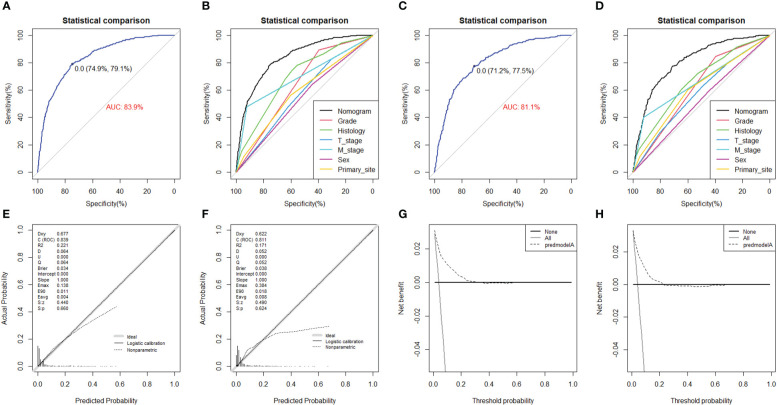
The ROC curve in the training set **(A)** and the testing set **(C)**, comparison of AUC between diagnostic nomogram and all predictors in the training set **(B)** and the testing set **(D)**. The calibration curve in the training set **(E)** and the testing set **(F)**, and DCA curve in the training set **(G)** and the testing set **(H)**.

**Figure 5:**
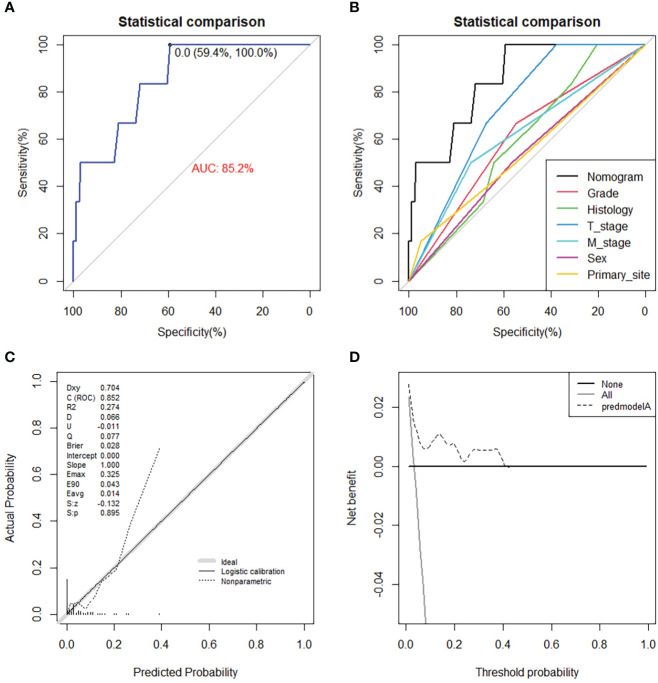
The ROC curve **(A)**, comparison of AUC between diagnostic nomogram and all predictors **(B)**, calibration curve **(C)**, and DCA curve **(D)** in the external testing set from Chinese population.

On the basis of six independent risk factors, a diagnostic nomogram was constructed to predict the probability of LNM in patients with STS (**Figure 3**). The nomogram showed that histology and M stage contributed the most to the occurrence of LNM. To promote the clinical application of this novel predictive model, we further developed a web-based probability calculator and a corresponding QR code (https://tyxupup.shinyapps.io/probabilityofLNMforSTSpatients/). The AUC values of the nomogram in the training set, the testing set, and the external testing set were 0.839 (95% CI, 0.821–0.857), 0.811 (95% CI, 0.783–0.840), and 0.852 (95% CI, 0.717–0.988), which indicated a good discrimination of the nomogram (**Figures 4A, C**, **5A**). Furthermore, we also generated ROC curves for each independent risk factor and compared the AUC values between nomogram and single predictor (**Figures 4B, D**, **5B**). These results suggested that the discriminative ability of nomogram was significantly better than each independent risk factor in both the training and testing sets. The calibration curves showed a good consistence between logistic calibration outcomes and predicted outcomes, which demonstrated a good calibration of the diagnostic nomogram (**Figures 4E, F**, **5C**). In addition, the DCA curves demonstrated a preferable positive net benefit, which suggested its strong clinical utility (**Figures 4G, H**, **5D**).

### Independent prognostic factors for OS and CSS in patients with STS with LNM

Of all the enrolled patients with STS, 659 patients were confirmed to have LNM at the time of diagnosis and assigned in the prognostic study cohort. In addition, these patients were divided into the training set (N = 463) and the testing set (N = 196). The basic demographic, pathological, and treatment characteristics of the patients with STS with LNM in two sets are listed in **Table 4**. Among these patients, 417 (63.28%) patients underwent surgery, 304 (46.13%) patients underwent LND, 318 (48.25%) patients underwent radiotherapy, and 395 (59.94%) patients underwent chemotherapy. On the basis of the results of univariate and multivariate Cox regression analysis, eight variables were identified to be the independent prognostic factors for OS: age, M stage, histology, grade, T stage, chemotherapy, radiotherapy, and surgery (P < 0.05) (**Table 5**). In terms of CSS, age, T stage, M stage, grade, radiotherapy, and surgery were the independent prognostic factors (**Table 6**).

**Table 4:** The baseline characteristics of patients with soft tissue sarcoma with lymph node metastasis.

Variables	Overall cohort (N = 659, %)	Training set (N = 463, %)	Testing set (N = 196, %)	P-value
**Age**				0.8142
<65 years	431 (65.40)	301 (65.01)	130 (66.33)	
≥65 years	228 (34.60)	162 (34.99)	66 (33.67)	
**Sex**				0.0675
Female	245 (37.18)	183 (39.52)	62 (31.63)	
Male	414 (62.82)	280 (60.48)	134 (68.37)	
**Race**				0.6481
Black	89 (13.51)	59 (12.74)	30 (15.31)	
Other	55 (8.35)	38 (8.21)	17 (8.67)	
White	515 (78.15)	366 (79.05)	149 (76.02)	
**Marital status**				0.1154
Married	305 (46.28)	224 (48.38)	81 (41.33)	
Unmarried	354 (53.72)	239 (51.62)	115 (58.67)	
**Primary site**				0.0167
Extremity	280 (42.49)	184 (39.74)	96 (48.98)	
Trunk	305 (46.28)	231 (49.89)	74 (37.76)	
Head and neck	74 (11.23)	48 (10.37)	26 (13.27)	
**Grade**				
Well differentiation	80 (12.14)	54 (11.66)	26 (13.27)	0.6561
Poor differentiation	579(87.86)	409 (88.34)	170 (86.73)	
**Histology**				0.3355
Fibrosarcoma	63 (9.56)	49 (10.58)	14 (7.14)	
Liposarcoma	43 (6.53)	29 (6.26)	14 (7.14)	
Leiomyosarcoma	61 (9.26)	42 (9.07)	19 (9.69)	
Synovival sarcoma	36 (5.46)	24 (5.18)	12 (6.12)	
MPNST	24 (3.64)	22 (4.75)	2 (1.02)	
Rhabdomyosarcoma	65 (9.86)	44 (9.50)	21 (10.71)	
Hemangiosarcoma	40 (6.07)	27 (5.83)	13 (6.63)	
Other	327 (49.62)	226 (48.81)	101 (51.53)	
**T stage**				0.2459
T1	117 (17.75)	90 (19.44)	27 (13.78)	
T2	229 (34.75)	159 (34.34)	70 (35.71)	
T3	168 (25.49)	119 (25.70)	49 (25.00)	
T4	145 (22.00)	95 (20.52)	50 (25.51)	
**M stage**				0.1354
M0	359 (54.48)	243 (52.48)	116 (59.18)	
M1	300 (45.52)	220 (47.52)	80 (40.82)	
**Surgery**				0.7942
No	242 (36.72)	172 (37.15)	70 (35.71)	
Yes	417 (63.28)	291 (62.85)	126 (64.29)	
**Radiotherapy**				0.6185
None	341 (51.75)	243 (52.48)	98 (50.00)	
Yes	318 (48.25)	220 (47.52)	98 (50.00)	
**Chemotherapy**				0.8593
No	264 (40.06)	187 (40.39)	77 (39.29)	
Yes	395 (59.94)	276 (59.61)	119 (60.71)	
**LND**				0.4007
No	355 (53.87)	244 (52.70)	111 (56.63)	
Yes	304 (46.13)	219 (47.30)	85 (43.37)	

MPNST, malignant peripheral nerve sheath tumor; LND, lymph node dissection.

**Table 5:** Univariate and multivariate Cox regression analysis for identification independent prognostic factors for overall survival in patients with soft tissue sarcoma with lymph node metastasis.

Variables	Univariate analysis		Multivariate analysis	
	HR (95% CIs)	P-value	HR (95% CIs)	P-value
**Age**				
<65 years	Reference		Reference	
≥65 years	1.76 (1.42–2.19)	<0.001	1.96 (1.50–2.55)	<0.001
**Sex**				
Female	Reference			
Male	0.96 (0.78–1.20)	0.743		
**Race**				
Black	Reference			
Other	0.98 (0.60–1.58)	0.922		
White	0.99 (0.71–1.36)	0.931		
**Marital status**				
Married	Reference			
Unmarried	0.94 (0.76–1.16)	0.566		
**Primary site**				
Extremity	Reference		Reference	
Trunk	1.25 (1.00–1.56)	0.055	0.93 (0.72–1.19)	0.5568
Head and neck	0.66 (0.44–0.99)	0.044	0.82 (0.53–1.27)	0.3686
**Grade**				
Well differentiation	Reference			
Poor differentiation	1.69 (1.17–2.45)	0.005	2.12 (1.42–3.17)	<0.001
**Histology**				
Fibrosarcoma	Reference		Reference	
Liposarcoma	0.77 (0.44–1.35)	0.367	0.72 (0.40–1.32)	0.2938
Leiomyosarcoma	0.88 (0.54–1.44)	0.619	0.83 (0.49–1.38)	0.4645
Synovival sarcoma	0.56 (0.29–1.05)	0.069	1.02 (0.52–1.97)	0.9634
MPNST	0.95 (0.53–1.72)	0.874	1.21 (0.65–2.28)	0.546
Rhabdomyosarcoma	0.52 (0.31–0.88)	0.015	0.63 (0.36–1.11)	0.1098
Hemangiosarcoma	1.44 (0.86–2.42)	0.167	2.21 (1.28–3.82)	0.0045
Other	1.29 (0.90–1.85)	0.165	1.12 (0.77–1.63)	0.556
**T stage**				
T1	Reference		Reference	
T2	1.76 (1.26–2.44)	0.001	1.72 (1.21–2.46)	0.0026
T3	2.08 (1.47–2.93)	<0.001	2.20 (1.50–3.23)	<0.001
T4	2.44 (1.72–3.46)	<0.001	2.63 (1.76–3.91)	<0.0010
**M stage**				
M0	Reference		Reference	
M1	2.41 (1.94–3.00)	<0.001	2.28 (1.74–2.99)	<0.001
**Surgery**				
No	Reference		Reference	
Yes	0.41 (0.33–0.51)	<0.001	0.42 (0.32–0.55)	<0.001
**Radiotherapy**				
No	Reference		Reference	
Yes	0.72 (0.58–0.89)	0.003	0.77 (0.61–0.97)	0.0246
**Chemotherapy**				
No	Reference		Reference	
Yes	0.75 (0.61–0.94)	0.01	0.53 (0.41–0.68)	<0.001
**LND**				
No	Reference		Reference	
Yes	0.61 (0.5–0.76)	<0.001	1.11 (0.85–1.45)	0.4328

MPNST, malignant peripheral nerve sheath tumor; LND, lymph node dissection; HR, hazard ratio; CIs, confidence intervals.

**Table 6:** Univariate and multivariate Cox regression analysis for identification independent prognostic factors for cancer-specific survival in patients with soft tissue sarcoma with lymph node metastasis.

Variables	Univariate analysis		Multivariate analysis	
	HR (95% CIs)	P-value	HR (95% CIs)	P-value
**Age**				
<65 years	Reference		Reference	
≥65 years	1.63 (1.29–2.05)	<0.001	2.19 (1.72–2.79)	<0.001
**Sex**				
Female	Reference			
Male	0.98 (0.78–1.24)	0.888		
**Race**				
Black	Reference			
Other	1.08 (0.65–1.80)	0.771		
White	1.09 (0.77–1.55)	0.624		
**Marital status**				
Married	Reference			
Unmarried	0.93 (0.75–1.17)	0.554		
**Primary site**				
Extremity	Reference			
Trunk	1.24 (0.98–1.57)	0.074		
Head and neck	0.67 (0.44–1.03)	0.067		
**Grade**				
Well differentiation	Reference			
Poor differentiation	1.64 (1.12–2.41)	0.011	1.77 (1.2–2.60)	0.0036
**Histology**				
Fibrosarcoma	Reference			
Liposarcoma	0.73 (0.40–1.34)	0.309		
Leiomyosarcoma	0.89 (0.54–1.49)	0.668		
Synovival sarcoma	0.53 (0.27–1.05)	0.067		
MPNST	0.94 (0.50–1.76)	0.85		
Rhabdomyosarcoma	0.59 (0.35–1.01)	0.053		
Hemangiosarcoma	1.41 (0.81–2.44)	0.225		
Other	1.35 (0.92–1.97)	0.123		
**T stage**				
T1	Reference		Reference	
T2	1.70 (1.20–2.40)	0.003	1.41 (0.99–2.01)	0.0595
T3	2.17 (1.51–3.11)	<0.001	1.77 (1.22–2.58)	0.0029
T4	2.48 (1.72–3.58)	<0.001	2.31 (1.57–3.40)	<0.001
**M stage**				
M0	Reference		Reference	
M1	2.50 (1.99–3.14)	<0.001	2.09 (1.60–2.72)	<0.001
**Surgery**				
No	Reference		Reference	
Yes	0.39 (0.31–0.49)	<0.001	0.48 (0.37–0.63)	<0.001
**Radiotherapy**				
No	Reference		Reference	
Yes	0.73 (0.58–0.92)	0.006	0.78 (0.62–0.98)	0.0345
**Chemotherapy**				
No	Reference		Reference	
Yes	0.75 (0.61–0.94)	0.01	0.60 (0.47–0.77)	<0.001
**LND**				
No	Reference		Reference	
Yes	0.59 (0.47–0.74)	<0.001	1.07 (0.82–1.40)	0.6162

MPNST, malignant peripheral nerve sheath tumor; LND, lymph node dissection; HR, hazard ratio; CIs, confidence intervals.

### Construction and validation of prognostic nomogram

**Figure 6:**
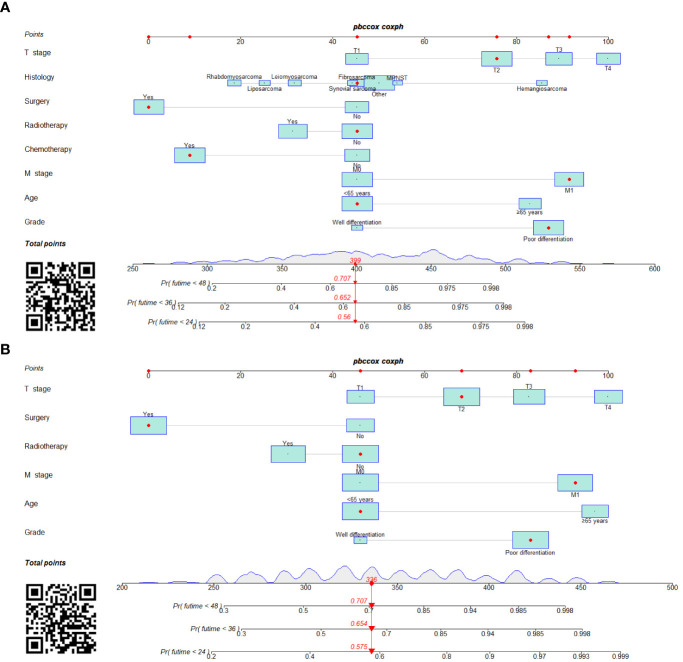
Prognostic nomograms in predicting 24-, 36-, and 48-month OS **(A)** and CSS **(B)** for patients with STS with LNM.

**Figure 7:**
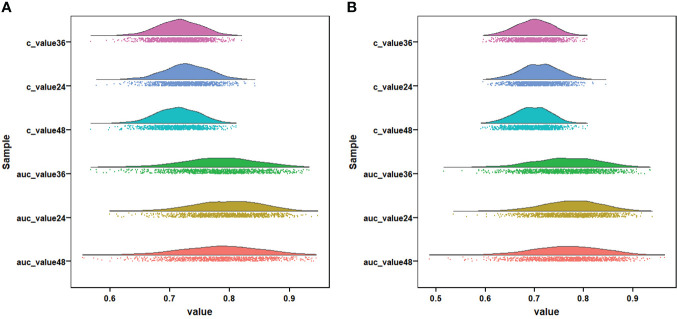
Visualization of the result of k-fold cross-validation (k = 10) through with half violin plot, scatter plot, and boxplot with median. Part **(A)** is for OS analysis and part **(B)** is for CSS analysis.

**Figure 8:**
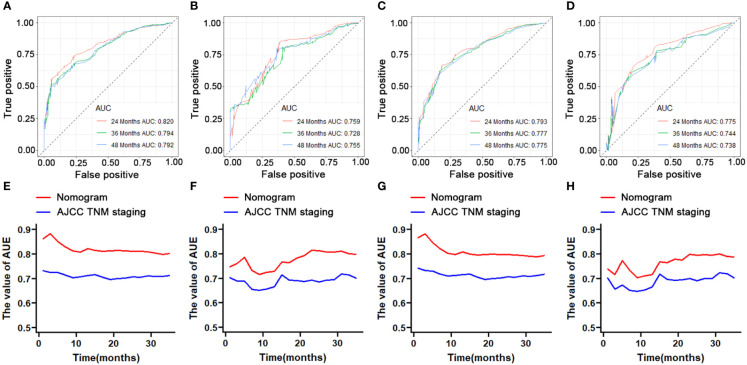
ROC curve analysis of the nomogram for the 24-, 36-, and 48-month in the training set (part **A**, OS; part **C**, CSS) and the testing set (part **B**, OS; part **D**, CSS). The time-dependent ROC curves for comparison of the discriminative ability between nomogram and TNM staging system in the training set (part **E**, OS; part **G**, CSS) and testing set (part **F**, OS; part **H**, CSS).

**Figure 9:**
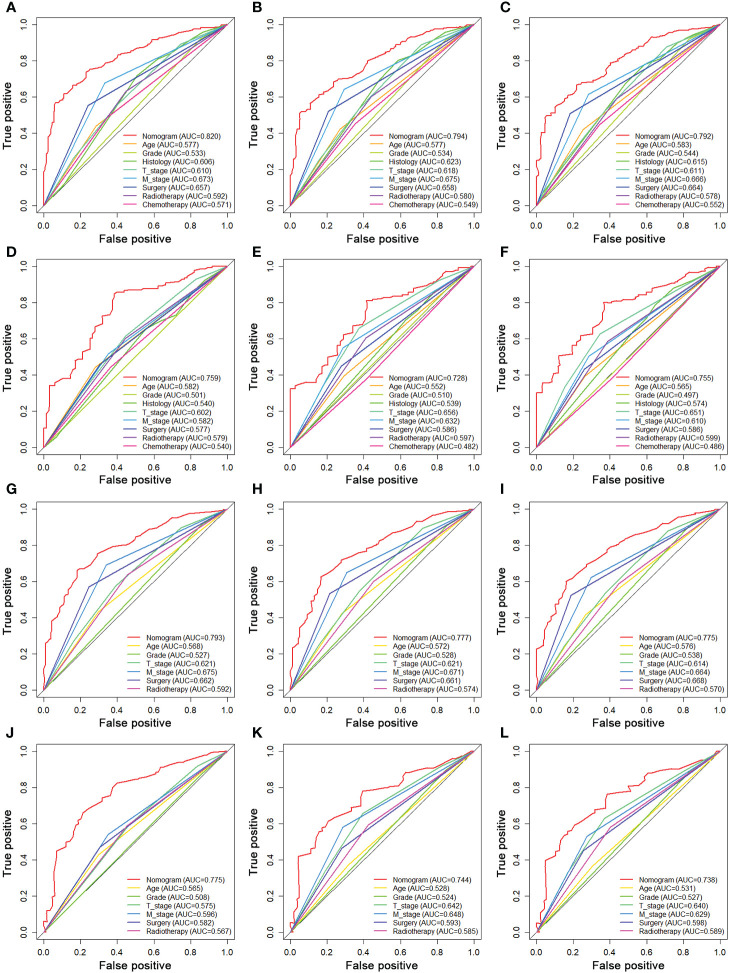
Comparison of AUC between prognostic nomogram and all predictors of 24- **(A)**, 36- **(B)**, and 48-month **(C)** OS in the training set and of 24- **(D)**, 36- **(E)**, and 48-month **(F)** OS in the testing set. Comparison of AUC between prognostic nomogram and all predictors of 24- **(G)**, 36- **(H)**, and 48-month **(I)** CSS in the training set and of 24- **(J)**, 36- **(K)**, and 48-month **(L)** CSS in the testing set.

**Figure 10:**
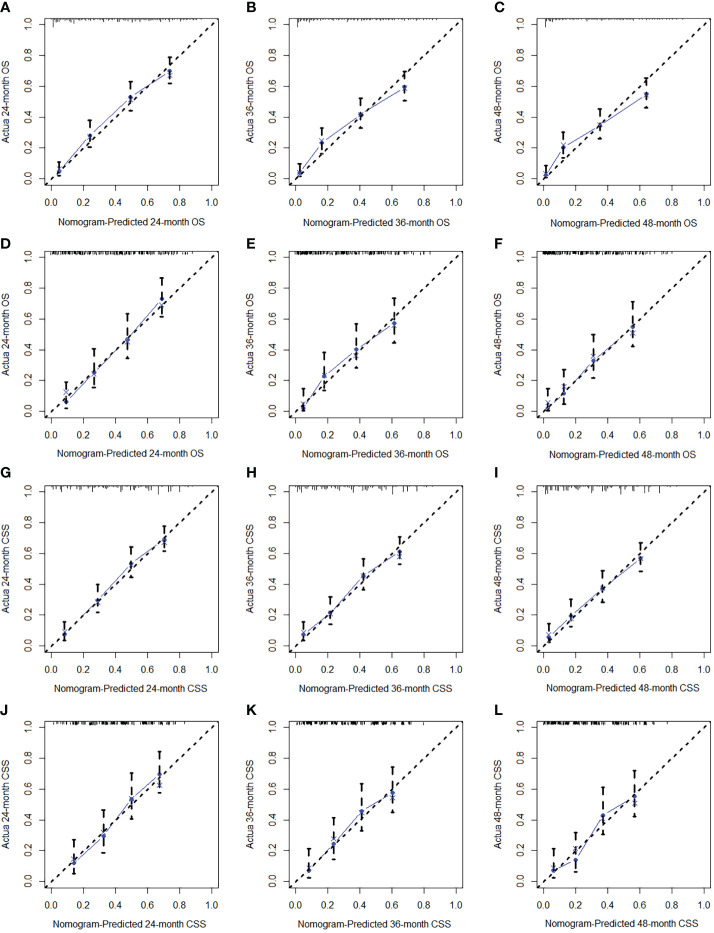
The calibration curves of 24- **(A)**, 36- **(B)**, and 48-month **(C)** OS in the training set and 24- **(D)**, 36- **(E)**, and 48-month **(F)** OS in the testing set. The calibration curves of 24- **(G)**, 36- **(H)**, and 48-month **(I)** CSS in the training set and 24- **(J)**, 36- **(K)**, and 48-month **(L)** CSS in the testing set.

**Figure 11:**
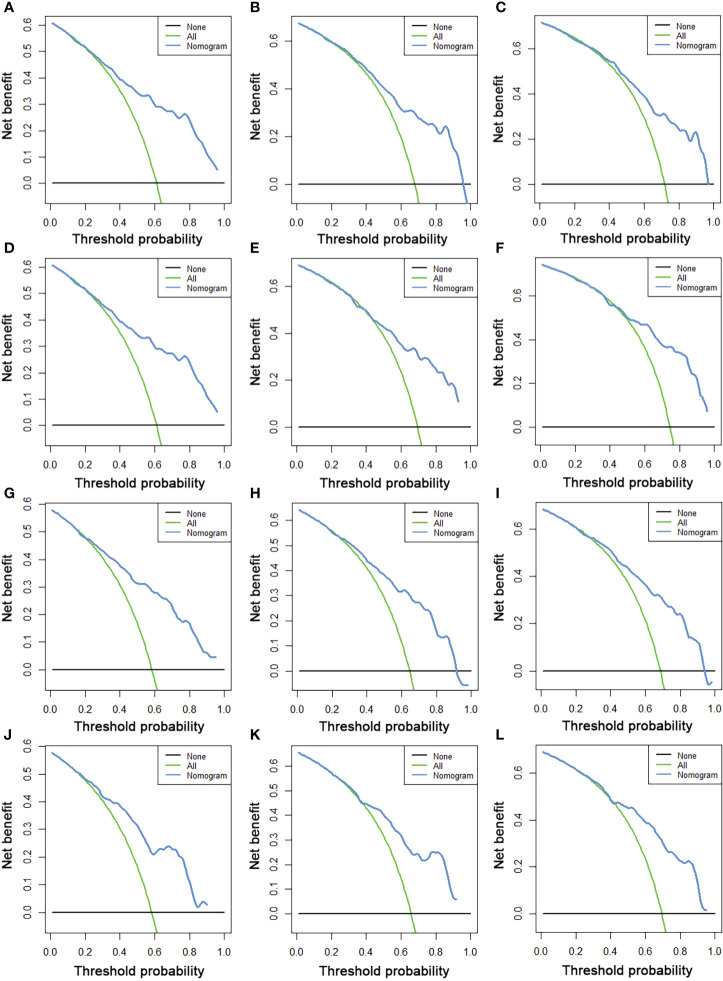
The DCA curves of 24- **(A)**, 36- **(B)**, and 48-month **(C)** OS in the training set and 24- **(D)**, 36- **(E)**, and 48-month **(F)** OS in the testing set. The DCA curves of 24- **(G)**, 36- **(H)**, and 48-month **(I)** CSS in the training set and 24- **(J)**, 36- **(K)**, and 48-month **(L)** CSS in the testing set.

We constructed two novel prognostic nomograms and their online version (https://tyxupup.shinyapps.io/OSofSTSpatientswithLNM/ and https://tyxupup.shinyapps.io/CSSofSTSpatientswithLNM/) to predict 24-, 36-, and 48-month OS and CSS probability of patients with STS with LNM (**Figure 6**). The total score for a patient with STS with LNM can be calculated by summing each point, and then, the survival probability at 24-, 36-, and 48-month could be determined. For example, the total score of a 50 year-old patient with fibrosarcoma with poor differentiation of primary tumor at M1 stage and T2 stage, who underwent chemotherapy and surgery but did not underwent radiotherapy, was 399 after summing each point. Hence, the OS probabilities at 24-, 36-, and 48-month were 0.560, 0.652, and 0.707, respectively. The result of k-fold cross validation (k = 10) indicated that the values of AUC for 24-, 36-, and 48-month OS were 0.795, 0.782, and 0.784, and values of AUC for 24-, 36-, and 48-month CSS were 0.775, 0.764, and 0.766 (**Figure 7**). In the training set, the nomogram for OS had AUC values of 0.820, 0.794, and 0.792 at 24-, 36-, and 48-month, respectively, and the nomogram for CSS had AUC values of 0.793, 0.777, and 0.775 at 24-, 36-, and 48-month, respectively. Meanwhile, in the testing set, the nomogram for OS had AUC values of 0.759, 0.728, and 0.755 at 24-, 36-, and 48-month, respectively. In addition, the nomogram for CSS had AUC values of 0.775, 0.744, and 0.738 at 24-, 36-, and 48-month, respectively (**Figures 8A–D**). The time-dependent ROC curves in two sets both showed that the discrimination ability of the nomogram was better than the AJCC TNM staging system (**Figures 8E–H**). We further compared the AUC values of each independent predictor and the comprehensive model at 24-, 36-, and 48-month (**Figure 9**). These results, taken together, indicated that the survival prediction based on the novel nomogram was more accurate than based on single prognostic factors and the conventional AJCC TNM staging system. The calibration curves for 24-, 36-, and 48-month OS and CSS in both two sets showed good agreements between the predicted and actual outcomes, which demonstrated a good calibration of two prognostic nomograms (**Figure 10**). In addition, the DCA curves in the training and testing sets showed positive net benefit across a wide range of death risks, suggesting a favorable clinical usefulness of the nomogram in predicting 24-, 36-, and 48-month OS and CSS probability (**Figure 11**).

**Figure 12:**
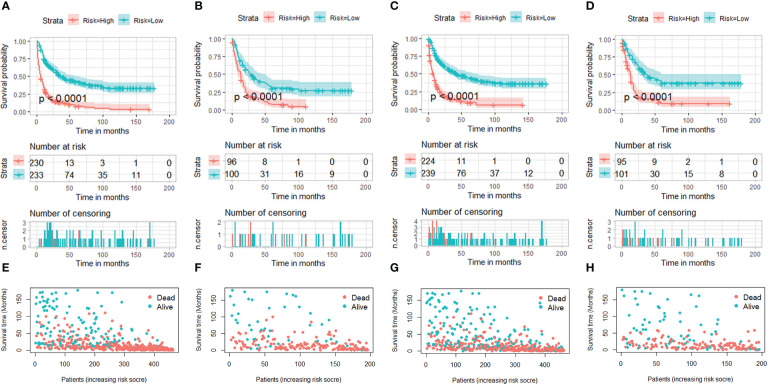
Kaplan–Meier survival curves of two mortality risk subgroups in the training set (part **A**, OS; part **C**, CSS) and testing set (part **B**, OS; part **D**, CSS). Kaplan–Meier survival status analysis for patients with STS with LNM in the training set (part **E**, OS; part **G**, CSS) and testing set (part **F**, OS; part **H**, CSS).

In addition, the total scores for each patient with STS with LNM were calculated on the basis of the prognostic nomograms. These patients were divided into high-risk and low-risk subgroups for K-M survival analysis. The results indicated that patients in the low-risk subgroup had better OS and CSS probability than patients in the high-risk subgroup (**Figure 12**).

## Discussion

It is generally assumed that distant metastases from STS are most commonly metastasized to the lungs *via* a hematogenous route (17). Although the incidence of LNM is relatively low, the survival probability of patients with STS with LNM is significantly worse than those without LNM (18, 19). In addition, nodal involvement is considered as marker of disseminated disease rather than regional disease because the development of LNM usually indicates biological aggressiveness of the primary tumor and is associated with the coexistence of occult micrometastatic disease, which is eventually responsible for the concomitant or subsequent DM (20, 21). Our findings did echo these reports from previous studies, where the probability of DM was just less than 10% in the entire cohort of patients with STS, whereas metastatic disease was present in nearly half cases of the cohort of patients with STS with LNM (**Tables 1**, **4**). Several recent studies have suggested that LNM was significantly related to overall poor outcome and was determined to be an independent negative prognostic factor predicting shorter 5-year overall survival of patients with STS (22–24). As shown in **Figure 2**, the results of K-M survival analysis in our study were also consistent with previous reports. These facts underscored that the attention on LNM in patients with STS should be more paid in clinical practice, and it was of particular importance to develop effective models to stratify the risk of LNM in patients with STS and predict survival rate of patients with STS with LNM. For those patients with STS at high-risk of LNM, 18F-FDG PET imaging should be more recommended this population for the purpose of early detection the LNM (25).

The earliest retrospective study of LNM in STS was performed by Weingrad and colleagues of the National Cancer Institute over a 24-year period, in which they included 374 cases of STS and found that rhabdomyosarcoma and synovial sarcoma were associated with an increased risk of LNM (26). In addition, in 1987, a cohort study implemented by Mazeron et al. showed that patients with STS with LNM tended to be poorly differentiation, rhabdomyosarcoma histology, and epithelioid histology (27). A more recent research also suggested that grade, histology, and tumor size were found to be correlated with the occurrence of LNM in patients with STS (7). Nevertheless, it was to be noted that previous studies were often small sample size and primarily based on single institution. Another population-based analysis from Liu et al. focused on exploring the independent risk and prognostic factors for patients with STS with LNM. Nevertheless, most previous studies, including the study by Liu et al., stopped at investigation of single LNM-related factor rather than visualizing of the risk of LNM, which made it difficult to inform orthopedic surgeons of the specific probability of LNM in patients with STS in clinical practice. To address this inadequacy, we incorporated the latest large samples with comprehensive clinicopathological data from the SEER database and found that the incidence of LNM was 3.97%. Six significant predictors for LNM in patients with STS were identified, namely, histology, grade, age, T stage, M stage, and primary site. Afterward, we developed a diagnostic nomogram to quantify the probability of LNM in patients with STS, which may improve the current situation of risk assessment of LNM and guide the individualized medical decision-making and clinical practice. It was worth mentioning that this novel nomogram was also well validated using the data of an external testing set from Asian population, which demonstrated the good extrapolation and general applicability of the model, which would make our prediction results more convincing. The correlation between histological type and LNM in patients with STS has been confirmed in various published studies (12, 28, 29). In our study, rhabdomyosarcoma was considered the histological type with the highest risk of LNM. The variation in risk of nodal disease by histological type may be related to the different biological features of STS differentiation from histology. Moreover, those patients with STS with poorly differentiated primary tumor and the presence of DM tended to have a higher risk of LNM. Tumor grade was classified on the basis of histological characteristics including histologic subtype, tumor necrosis, and mitotic activity (30). An early study suggested that 45 of the 46 patients with LNM from various forms of STS were of high grade (31). Another study implemented by Behranwala et al. investigated 2,127 cases of STS and found a 70% association between involvement of regional lymph nodes and high-grade tumors. It was also reported that a larger tumor size frequently indicated a greater biological aggressiveness and more likely to spread to regional lymph nodes, and this was also confirmed by our findings (32, 33). In addition, in contrast to other anatomical regions, the lymphatic channels in the head and neck were greater density, which might explain why patients with STS of the head and neck were more likely to develop LNM (34). In terms of demographic characteristics, we found that male patients and elderly patients were prone to suffer from nodal disease. Aging is a continuous and comprehensive process that is accompanied by a progressive decline of the immune system and cellular aging, including changes in proteins, metabolism, and nuclear genomic instability (35, 36), which may be involved in progression of tumors.

Furthermore, we also found that those patients with STS with LNM with characteristics such as advanced age, M1 stage, higher T stage, poorer differentiated primary tumor, and not receiving surgery, radiotherapy, and chemotherapy had worse survival outcome. A previous study showed that more than half of patients with extremity STS with LNM (52.1%) had concomitant DM, and the prognosis of patients with STS with N1M1 stage was poorer than those with N1M0 stage (37). Moreover, older age (≥65 years) was also confirmed to be associated with worse OS and CSS in patient with STS with LNM. The underlying reasons may be that elderly patients tended to have various underlying diseases, such as cardiovascular disease, respiratory diseases, and diabetes (38). In addition, because of their poor physical condition, elderly patients frequently had poorer therapy response rates and higher rate of surgery-related complications (39, 40). Moreover, Basile et al. suggested that those patients with STS with LNM who were actively treated with anti-tumor therapy had more satisfactory survival outcome (41). In our study, we confirmed that surgery could provide significant survival benefit in patients with STS with LNM. Danna et al. demonstrated that surgical resection of primary tumor could reverse the immunosuppressive effects caused by tumor progression (42). In addition, another study assumed that removing of tumor stem cells from the primary site would reduce the production of drug-resistant cell lines (43). Tumor self-seeding theory also might explain the positive role of primary tumor surgery. Nevertheless, we noted that only about 60% of patients received surgical treatment, which means that probably 40% of the cohort of patients with STS with LNM was not treated with radical intent. Combining our findings with previous reports in the literature, it is therefore necessary to actively consider surgical treatment for patients with STS with LNM. In addition, because STS with LNM was defined as AJCC IV stage, chemotherapy was deemed as a treatment option for patients at this stage. In patients with metastatic STS, anthracyclines, either alone or in combination with ifosfamide, was recommended as a standard first-line therapy (44). Moreover, there is an increasing evidence that patients with metastatic STS might benefit from radiotherapy (45). In addition, our results showed that radiotherapy could improve OS rates of patients with STS with LNM. This finding was agreed with a recent retrospective study implemented by Qiu et al., in which they investigated 265 patients and found compared to surgery alone, surgery combined with radiotherapy could improve the CSS and OS of patients with extremity STS with LNM (46). On the basis of abovementioned identified prognostic factors, two web-based prognostic nomograms were developed to predict the OS and CSS rate of patients with STS with LNM, and the excellent predictive performance was validated by calibration curves, ROC curves, and DCA, which would help to fill the gap in survival prediction for patients with STS with LNM and contribute to optimal clinical management.

At present, the potential benefit of LND remains controversial. Several previous studies have studied the effect of LND and suggested that it could lengthen survival time of patients with STS with LNM (31, 47). Nevertheless, although the univariate Cox analysis suggested that LND was potentially associated with improved clinical outcome of patients with STS with LNM, the results from multivariate Cox analysis showed that LND was not the independent prognostic factor for OS and CSS. Similarly, Johannesmeyer et al. reported that, although the existence of LNM affected the prognosis, the degree of lymph node load and the degree of resected lymph nodes did not affect the survival probability (48). In addition, Sawamura et al. suggested that LND could improve short-term survival but might not bring substantial survival benefits in the long term in patients with STS with LNM (49). In a study conducted by Outani and colleagues, of the eight patients with epithelioid sarcomas with LNM receiving lymphadenectomy, four developed new nodal metastasis at an average of 14 months, and three of these patients died of the disease. Lymphadenectomy did not confer a survival benefit in the patients who developed nodal metastasis (p = 0.611) (50). In addition, another study also did not recommend a policy of indiscriminate prophylactic nodal dissection (51). Therefore, further prospective studies are needed to assess the potential of LND.

There were several limitations in this study. First, this work was a retrospective study, and selection bias inevitably existed. Second, detailed treatment information and some other potentially relevant variables were not recorded in the SEER database. Future research could add tumor markers and gene expression variables to this base to develop a more comprehensive and superior predictive model. Finally, although the robust extrapolation of diagnostic nomogram had been confirmed with data from another cohort in Asia, because of the rarity of LNM in patients with STS, two prognostic nomograms were still not externally validated in the clinical setting; thus, their clinical value in other populations was unknown and the information derived from those should be used with caution.

## Conclusion

In this study, we determined the independent risk factors and the prognostic factors of patients with STS with LNM and developed three web-based nomograms to identify patients with STS at high risk of LNM and then to estimate survival outcome for these patients, which might help to guide clinical practice. Further testing and validation based on the data from large multicenter prospective studies were needed to confirm the generalization capability of prognostic nomograms in clinical application.

## Data availability statement

The raw data supporting the conclusions of this article will be made available by the authors, without undue reservation.

## Ethics statement

Ethics approval was exempted by the local ethics committee and the informed consent was also not required in this study.

## Author contributions

YT and DZ conceived of and designed the study. YT, YP, and YC collected the clinical data and literature review. YT conducted the statistical analysis. YT, LJ, YP, and YG generated the figures and tables. YT wrote the manuscript. YT and DZ revised the manuscript. DZ supervised the research. All authors critically read the manuscript to improve intellectual content. All authors read and approved the final manuscript.
